# Immune system-related soluble mediators and COVID-19: basic mechanisms and clinical perspectives

**DOI:** 10.1186/s12964-022-00948-7

**Published:** 2022-08-29

**Authors:** Mohammad Sadegh Soltani-Zangbar, Forough Parhizkar, Mojtaba Abdollahi, Navid Shomali, Leili Aghebati-Maleki, Sima Shahmohammadi Farid, Leila Roshangar, Ata Mahmoodpoor, Mehdi Yousefi

**Affiliations:** 1 Stem Cell Research Center, Tabriz University of Medical Sciences, Tabriz, Iran; 2 Student Research Committee, Tabriz University of Medical Sciences, Tabriz, Iran; 3 Department of Immunology, School of Medicine, Tabriz University of Medical Sciences, Tabriz, Iran; 4 School of Medicine, Urmia University of Medical Sciences, Urmia, Iran; 5 Immunology Research Center, Tabriz University of Medical Sciences, Tabriz, Iran; 6 Department of Anesthesiology, Faculty of Medicine, Tabriz University of Medical Sciences, Tabriz, Iran

**Keywords:** SARS-CoV-2, COVID-19, Soluble immune mediator, Soluble immune checkpoints

## Abstract

During SARS-CoV-2 infection, an effective immune response provides the first line of defense; however, excessive inflammatory innate immunity and impaired adaptive immunity may harm tissues. Soluble immune mediators are involved in the dynamic interaction of ligands with membrane-bound receptors to maintain and restore health after pathological events. In some cases, the dysregulation of their expression can lead to disease pathology. In this literature review, we described current knowledge of the basic features of soluble immune mediators and their dysregulation during SARS-CoV-2 infections and highlighted their contribution to disease severity and mortality.

**Video Abstract**

**Supplementary Information:**

The online version contains supplementary material available at 10.1186/s12964-022-00948-7.

## Introduction

Coronavirus disease 2019 (COVID-19) was officially announced as a pandemic since SARS-CoV-2 aggressively spread worldwide from December 2019 [1]. Its etiology is due to the severe acute respiratory syndrome coronavirus type 2 (SARS-CoV-2). The SARS-CoV-2 infection has been manifested in asymptomatic to severe symptoms such as acute respiratory distress syndrome (ARDS) and even death [2, 3]. The rapid and effective immune response against SARS-CoV-2 infection provides the first line of defense; however, excessive inflammatory innate immunity and impaired adaptive immune responses can harm localized and systemic tissues [4–6]. There is evidence that shows elevated levels of inflammatory and anti-inflammatory cytokines, including interferon (IFN)-γ, interleukin (IL)-6, IL-β, IL-8, and IL-10 in the COVID-19 patients with severe symptoms [7–9]. Notably, following SARS-CoV-2 infection, the massive release of cytokines and chemokine may occur, referred to as cytokine storm, demonstrating a widespread dysregulation of host immunity leading to multiorgan dysfunction [10, 11].

Based on the rapidly developing knowledge on the immune response against SARS-CoV-2, throughout this review, we will attempt to provide a comprehensive view of our current understanding of the other soluble mediators such as soluble immune checkpoints and cytokines receptors involved in the interaction between this virus and the host immunity. Furthermore, we will describe the role of soluble mediator abnormality in contributing to the pathogenicity and severity of COVID-19.

## Soluble immune checkpoints

Immune checkpoint molecules play an essential role in regulating the immune response. Transforming signals between immune cells can influence cell activity and cytokine secretion in response to the microenvironment. Besides the membrane-bound immune checkpoints, soluble checkpoints have also been discovered recently [12]. Soluble immune checkpoints, co-stimulatory, and co-inhibitory molecules can be detected in human plasma and either produced by proteolytic cleavage of membrane-bound forms or through alternative splicing, preserving the functional domain of membrane-bound isoform [13]. The immune responses are maintained through them. However, in some cases, the dysregulation of their plasma levels can contribute to disease pathology. Table 1 provides an overview of immune-related soluble mediators following SARS-CoV-2 infection briefly.

**Table 1:** Role of immune soluble mediators in COVID-19 infection

Soluble mediator	Biological role in COVID-19 infection	Output effect	References
sPDL-1	Decrease of effector T lymphocytes Increase in lower PaO2/FIO2 ratio and higher CRP concentration	Protective effect	[15–18, 21]
sPD-1	Increase effector T lymphocytes function	Adverse effect	[15, 19, 20]
sTim-3	Activation and exhaustion marker of T lymphocytes in chronic inflammation and viral infection like SARS-CoV-2 Negative and positive correlation with PaO2/FIO2 ratio and NT-ProNBT, respectively	Adverse effect	[19, 24–26]
sTNFRI and sTNFRII	Increase in COVID-19 cases Cleaved by ADAM17 in chronic inflammation Increased mortality risk in cardiovascular diseases Correlate with illness severity iv COVID-19 cases	Adverse effect	[28, 30–34]
sIL-2R	Increase in COVID-19 cases Cause to lymphopenia and reduced cell response to IL-2 Negative regulator of regulatory T lymphocytes, NK cells, and B lymphocytes Negative association with PaO2/FiO2 ratio Positive correlation with morbidity in COVID-19 cases	Adverse effect	[36–40]
sIL-6R	Role in stromal epithelial response to IL-6 Agonist of IL-6 which is cleaved by ADAM17 Increase in HIV, influenza A, and severe COVID-19 cases Cause to increased release of MCP-1 from endothelial cells in COVID-19 cases ( cause to hyper inflammation)	Adverse effect	[43–48]

sPDL-1, soluble programmed cell death ligand 1; sPD-1, soluble programmed cell death 1; sTim3, soluble T-cell immunoglobulin and mucin domain 3; sTNFRI&II, soluble tumor necrosis factor receptor 1&2; sIL6R, soluble interleukin 6 receptor

### Soluble programmed cell death protein-1 (sPD-1) and sPD-ligand1 (L1)

PD-1 and PD-L1 interactions inhibit effector functions, including cytokine release, cytotoxicity, T cell proliferation, and survival. In addition, it induces apoptosis in tumor-specific T cells [14] and improves CD4+ T-cells differentiation to Foxp3+ regulatory T cells [15]. PD-1 has been found to have four splice variants so that sPD-1 can be produced by alternatively spliced mRNA [16], while sPD-L1 is thought to be generated by proteolytic cleavage of a membrane-bound isoform of PD-L1 [17]. It has been shown that the parallel rise of both soluble PD-1 and PD-L1 molecules may have regulation properties to counteract each other's activities, such as membrane-bound upregulation [18]. sPD-1, through binding to mPD-L1, could interrupt membrane-bound PD-1 (mPD-1)/mPD-L1 interaction; thereby, the increased sPD-1 could prevent T cell inhibition. An increase in the level of sPD-L1 might also further inhibit T cell function, promoting tumor immune evasion and causing poor outcomes [19]. It has been found that sPD-L1 exerts immunosuppressive effects, either by inhibiting T cell activation or enhancing its apoptosis [20]. An abnormality in the level of sPD-1 and sPD-L1 has been recently indicated following SARS-CoV-2 infectious. So that, the amount of sPD-L1 is increased in COVID-19 patients as compared to healthy controls [21, 22], which is correlated with a lower number of lymphocytes and partial pressure of oxygen (PaO2) to the fraction of inspired oxygen (FIO2) (P/F) as well as a higher level of C reactive protein (CRP) [22]. Also, higher amounts of sPD-1 and sPD-L1 have been detected to be associated with the disease severity of COVID-19 [23, 24]. By contrast, a recent study revealed that the higher serum levels of sPD-L1 have a protective role in acute respiratory distress syndrome (ARDS), mostly associated with COVID-19. sPD-L1 can activate the PD-1 pathway. In this regard, as a result of sPD-L1 administration, inflammatory lung injury was effectively relieved, and the survival rate was improved in mice with direct ARDS, suggesting sPD-L1 as a promising agent in the recovery of COVID-19 patients with direct ARDS [25].

### Soluble T-cell immunoglobulin domain and mucin domain 3 (sTim-3)

Tim-3 is another reliable indicator of T cell exhaustion in disorders associated with persistent T cell activation, such as a chronic viral infection [26]. The soluble form of Tim-3 can be generated by proteolytic cleavage of membrane-bound isoform or spliced [27]. sTim-3 may have different properties depending on the type of construct production. So, surface shedding might increase T cell responses, although an alternatively spliced form of sTim-3 may inhibit them. It has been shown that recombinant sTim-3 mice can inhibit T cell responses to antigen-specific stimulation [28]. Thus, sTim-3 could be a valuable biomarker of persistent T cell activation and exhaustion in many conditions, including SARS-CoV-2 infections. An increase in the level of sTim-3 during acute COVID-19 infection could be considered as a marker of T cell activation, as suggested by the correlation between such molecule and sCD25 [29]. In addition, in this study, sTim-3 showed a negative correlation with the P/F ratio as a marker of respiratory failure and a positive correlation with N-terminal pro-B-type natriuretic peptide (NT-proBNP) as a cardiac marker. It suggests that T cells play a pathogenic role in the cardiac involvement associated with COVID-19 infection [29]. Severe COVID-19 condition is also characterized by elevated levels of sTim-3 [23, 29, 30], which indicates activation and potential exhaustion of T cells. This mechanism might prevent persistently and overactivation of T cells, which can adversely affect the host [29]. Moreover, a study found a negative correlation of sTIM-3 with absolute lymphocyte count [23].

### Other soluble immune checkpoints

The other soluble immune checkpoints are sCTLA-4, sLAG-3, sGITR, sBTLA, sHVEM, sCD28, sCD80, and sCD86, sCD27, sIDO, and s4-1BB have been reported in high levels in COVID-19 patients that were associated with disease severity. So, the levels of s4-1BB, sLAG-3, sIDO, sGITR, sCD28, and sCD27 negatively correlate with absolute CD4 and CD8 T lymphocytes count [23, 24].

## Soluble immune receptors

Soluble cytokine receptors can develop from genes encoding membrane-bound receptors or derived directly from receptors themselves. There is considerable evidence that soluble receptors are involved in the dynamic interaction of ligands with membrane-bound receptors to maintain and restore health after pathological events; however, in some cases, the dysregulation of soluble receptors' expression can lead to disease pathology [31].

### Soluble TNF receptors (sTNF-R1 and sTNF-R2)

TNF receptor 1 (TNFR1) is widely expressed in all body cells and lymphoid systems, which contributes to the wide-ranging function of TNF. While TNFR2 is expressed only by a subset of lymphocytes, including Tregs [32]. In general, TNF-a binding to TNFR1 induces apoptosis due to death domains, and by binding to TNFR2, cells survive. Although, some overlap may occur due to cell activation state and other factors [33]. The soluble form of membrane-bound (sTNFR1 and sTNFR2) is required for TNF-a signaling through various pathways. Cleavage of their transmembrane forms by ADAM17 results in a noticeable increase in serum levels of soluble TNF receptors [34]. The role of sTNFR1 and sTNFR2 is debated because they bind to TNF-α and prevent its action in acute inflammation. By contrast, in chronic inflammation, TNF-α-sTNFR1 complexes improve the function of TNF-a by slowing its release [32]. However, the concentration may influence these effects. In a previous study, it has been indicated that sTNFR1 and sTNFR2 are associated with mortality and increased risk of cardiovascular disease during advanced chronic kidney diseases, regardless of the cause of the kidney disease [35]. Furthermore, Nishiga et al. revealed cardiovascular diseases as a risk factor associated with enhanced mortality in patients with COVID-19 [36]. Recently, elevated serum levels of sTNFR1 and sTNFR2 have been found in severe COVID-19 patients related to mortality in patients in the Intensive care unit (ICU) [32, 37]. In addition, Bowman et al. found a higher level of sTNFR1 and sTNFR2 in critical patients who died than in those who recovered [38]. Moreover, a negative correlation was observed between the higher level of sTNFR1 and CRP, suggesting that it might activate pro-inflammatory mechanisms other than those mediated by CRP in severe patients [39].

### Soluble Interleukin (IL)-2 receptor (sCD25R)

IL-2R is expressed in various forms, including monomer, dimer, or trimer on immune cells, APCs, conventional T cells, and Tregs. Shedding of the IL-2R α-chain (CD25) results in a soluble form of IL-2R (sIL-2R). The binding of sIL-2R to IL-2 may decrease or increase responses depending on whether the target cell is involved in immunity or self-tolerance [40]. It has been shown that the circulating sIL-2R regulates the activation of T cells in various immunological diseases, and a higher concentration of sIL-2R in plasma indicates a diminished cell response to IL-2 [41]. Increased levels of sIL-2R have been shown in COVID-19 cases after disease onset, which could contribute to lymphopenia by inhibiting IL-2 signaling. It has been suggested that sIL-2R could be a negative regulator for T cells, particularly CD8+ T cells, but not CD4+ T cells, NK cells, or B cells [42]. Also, the shedding of sIL-2R in Treg cells can be regarded as a decoy receptor for IL-2 that inhibits T-cell responses, thereby preventing immune tolerance [40]. Studies indicate an association between sIL-2R and disease severity in COVID-19 patients [42]. So, the elevated serum level of sIL-2R was correlated with the P/F ratio, indicating the illness's severity [43]. Also, sIL-2R was meaningfully associated with mortality in COVID-19 patients suffering from respiratory failure, despite adjusting numerous variables [43, 44]. Based on the association between sIL-2R level and the clinical outcome of COVID-19 patients with respiratory failure, sIL-2R must be monitored sequentially, along with close observation.

### Soluble IL-6 receptor (sIL-6R)

IL-6 agonist receptor, which can take both transmembrane (IL-6R or gp80) and soluble forms (sIL-6R or gp50), bind to IL-6. As a result of this initial complex binding to gp130, intracellular signal transduction and gene expression initiate [45]. Stromal and epithelial cells don't express IL-6R, whereas they can respond to IL-6 by binding to the sIL-6R attached to IL-6 and then to the membrane gp130 receptor, triggering the trans-signaling pathway. Therefore, sIL-6R is an agonist of IL-6R and can enhance its function [46]. In addition to alternative splicing of IL-6R mRNA, most circulating sIL-6R arises from the ADAM-17-mediated cleavage of the transmembrane IL-6R [47, 48]. It is generally accepted that classic IL-6 signaling exerts anti-inflammatory effects while trans-signaling contributes to its pro-inflammatory properties [46]. A higher level of sIL-6R has been found in the serum of viral HIV-infected patients and those infected with the influenza A virus [49, 50]. Recently, an increased level of sIL-6R in patients infected with COVID-19 has been reported [51, 52]. In addition, the concentration of sIL-6R is high in severe COVID-19 but not correlated with IL-6 levels [53]. Following SARS-CoV-2 infection in epithelial cells, sIL-6R is released from these cells upon activation of ADAM-17 via SARS-CoV-2 spike protein [52]. Inducing IL-6 trans-signaling, chemokines, including monocyte chemoattractant protein-1 (MCP-1), from pulmonary vascular endothelial cells are released, and monocytes and macrophages are attracted to cause hyper inflammation, resulting in pulmonary edema, disruption of oxygen exchange, and ARDS [52].

## Neutrophil extracellular traps (NETs)

**Fig. 1:**
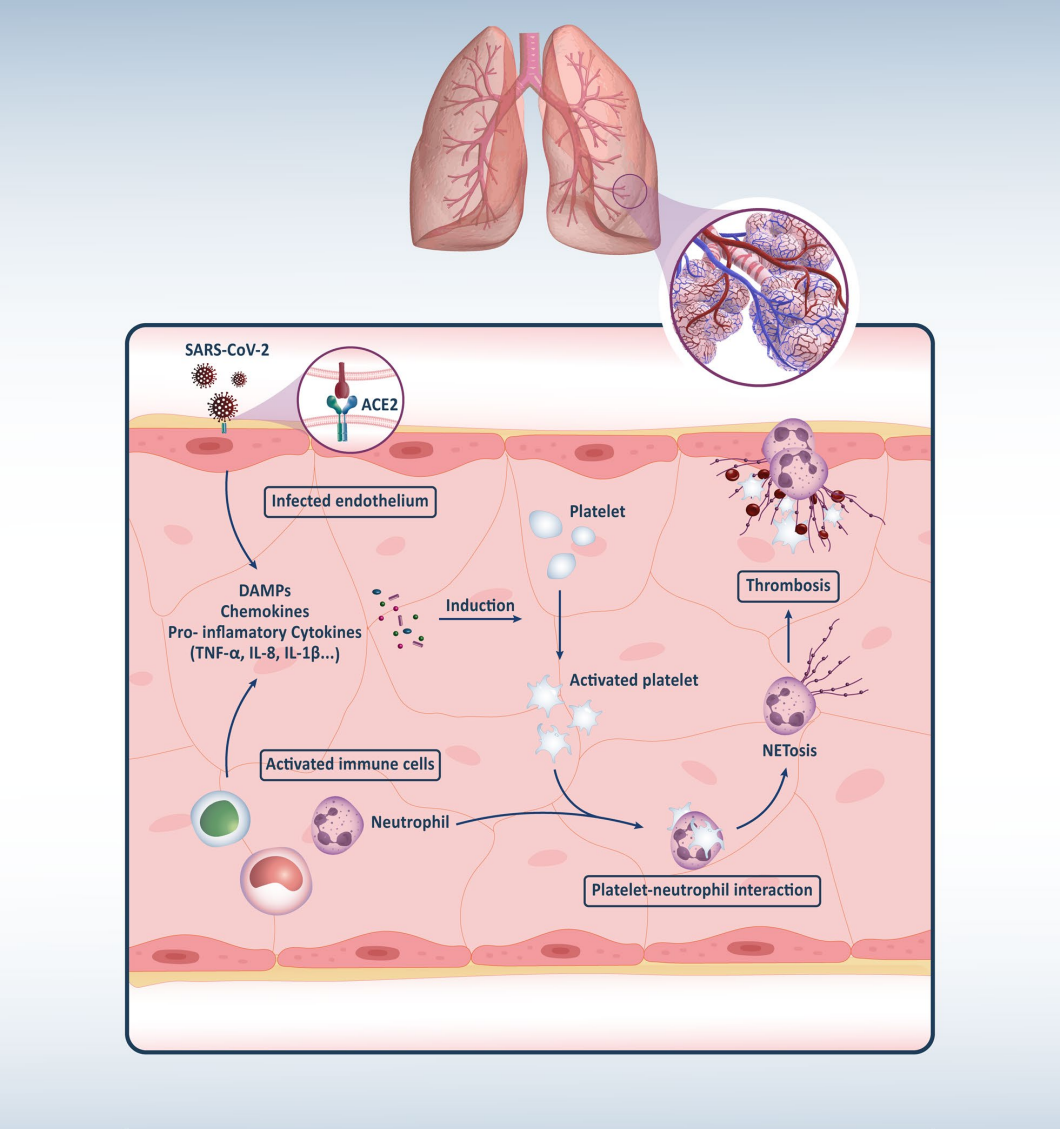
The possible pathway of neutrophil extracellular trap-osis (NETosis) in airway of COVID19 infected patients. Following infection of the lung airways with the SARS-CoV-2, neutrophils recruit and activates due to the secretion of proinflammatory cytokines, DAMPs, viral components, and platelets activations, and subsequently neutrophils undergo the process of NETosis to eliminate infectious agents. This action, in turn, causes more destruction of lung airways

NETs are extracellular lattices consisting of decondensed chromatin, histones, and antimicrobial proteins released by stimulation. Polymorphonuclear leukocytes, including PMNs and neutrophils, produce NETs and undergo a regulated cell death termed NETosis. Although NETs were initially recognized for their performance in bacterial clearance, they are now recognized as essential for innate immunity against a wide range of RNA-based viral pathogens, including influenza, respiratory syncytial disease, and HIV [54, 55]. In an infection, the production of NETs is not only triggered by microbes but also by pro-inflammatory mediators, including TNF-α, IL-8, auto-antibodies, and activated platelets [56]. Dysregulation of NETosis can also occur and result in hypercoagulability and tissue damage and correlate with acute and chronic inflammatory disorders [57]. Acute lung injury has been observed in SARS-CoV-2 infection and ARDS due to dysregulated NETosis triggered by respiratory viruses. Indeed, this condition is strongly influenced by the NET formation in the lung tissue and vasculature [58, 59]. Similar cases have also been found among other viral infections [60]. Middleton et al. confirm a higher level of myeloperoxidase (MPO)-DNA complexes in plasma from patients infected with COVID-19. So, the severe form of the disease is directly correlated with these complexes. In contrast, P/F are inversely correlated [57]. So, excessive Neutrophil activation and NETs formulation have been considered important factors affecting COVID-19 patients' mortality [57, 59]. In COVID-19 victims, NETs were also detected in the lung, heart, and kidney microvasculature, which contribute to immune thrombosis that induced these organ damages [61]. Figure 1 shows the process of NETosis following SARS-CoV-2 infection schematically. Based on these data, organ dysfunction in patients with severe COVID-19 is associated with the higher formation of NETs and vascular damage.

## Soluble platelet activation markers (sP-selectin and sCD40L)

**Fig. 2:**
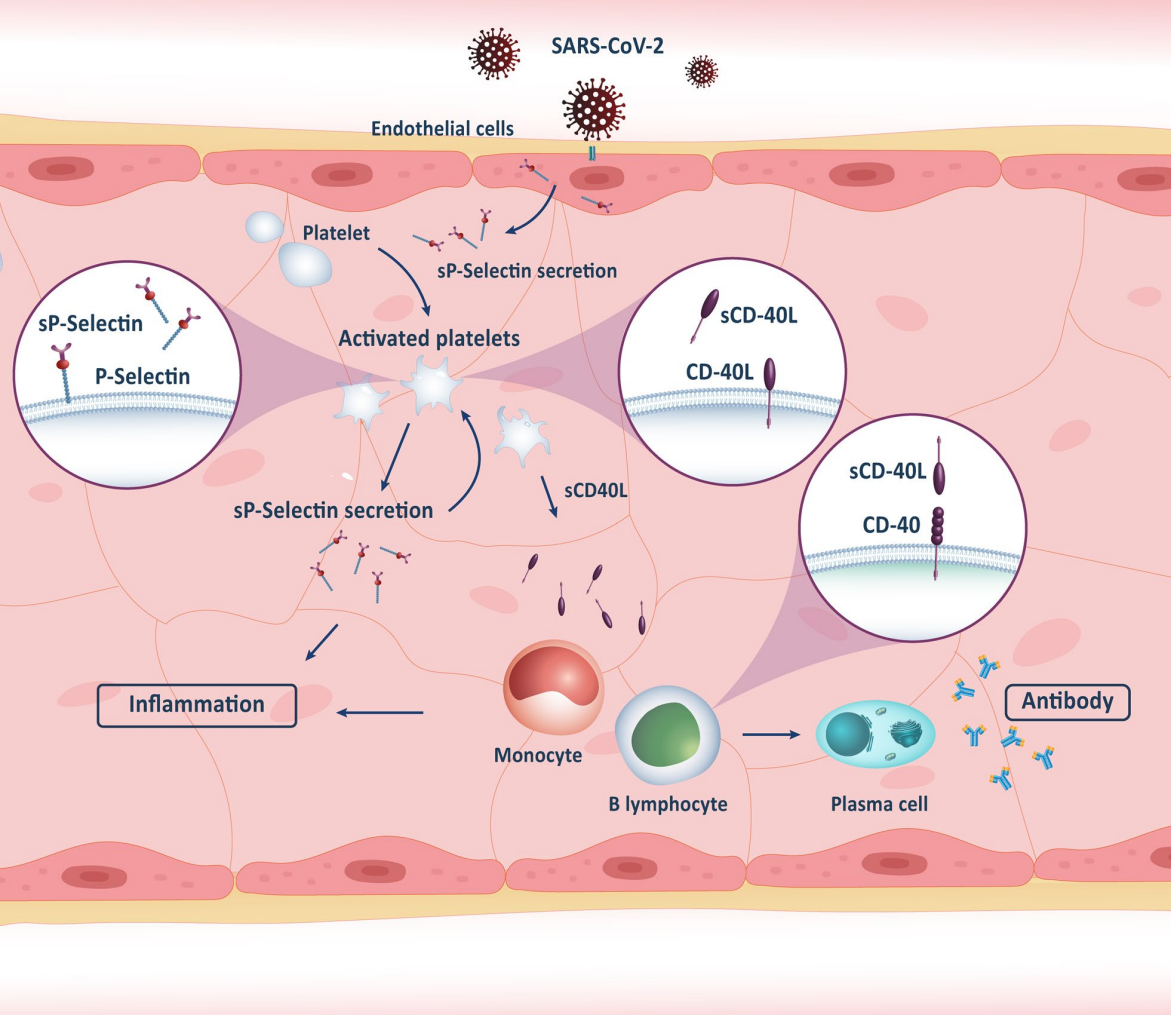
Inflammation mechanism based on the secretion of sP-selectin and sCD40L in SARS-CoV-2 infection. Following infection of the airways of the lungs with the SARS-CoV-2, endothelial cells secrete sP-selectins that cause recruitment and activation of the platelets. More secretion of sP-selectin and sCD40L by activated platelets cause more activation of platelets and monocytes, and activation and antibody production by B lymphocytes. Inflammation is the final result of the activated platelets, monocytes, and B lymphocytes via this pathway

The release of several biologically active molecules during vascular inflammation harms the endothelium and activates the platelets [62]. Soluble P-selectin (sP-selectin) is a molecule secreted by stimulated endothelial cells and platelets and interacts with white blood cells on the vascular surface [63]. It plays a crucial role in the inflammatory response during viral infections, such as influenza [64]. In addition, soluble CD40L (sCD40L) is another one that is mainly derived from activated platelets and provides vital signals for the production of immunoglobulin by B cells [65] and also has a significant role in shaping innate immunity through binding CD40 [62]. Recently, the potential role of such soluble molecules in the pathogenicity of SARS-CoV-2 has been investigated. Goshua et al. reported a higher level of sP-selectin and sCD40L in ICU patients with COVID-19 compared to the control group suggesting that SARS-CoV-2 induces the release of P-selectin and sCD40L from stimulated endothelial cells and triggers platelet activation. The elevated level of sP-selectin was also shown in ICU patients than in non-ICU [66], indicating these molecules' contribution to the severity of viral infection. Moreover, sP-selectin and sCD40L were correlated with thrombosis or mortality in patients hospitalized with COVID-19 [67, 68]. Figure 2 shows the inflammation process via sp-selectin and sCD40L following SARS-CoV-2 infection. Based on these studies, sP-selectin and sCD40L are involved in pathogenesis induced by SARS-CoV-2.

## Other soluble mediators

Other soluble mediators affect the immunological and physiological mechanisms of the body following SARS-CoV-2 infection. Table 2 provides these mediators with their effects in brief.

**Table 2:** Role of non-immune soluble mediators in COVID-19 infection

Soluble mediator	Biological role in COVID-19 infection	Output effect	References
sFlt-1	Excess level of sFlt-1 induce endothelial dysfunction and is associated with bacterial sepsis Increase in SARS-CoV-2 infection and is correlated with disease severity, endothelial dysfunction and respiratory failure	Adverse effect	[64–69]
sACE2	Increase in viral infections like SARS-CoV-2 Interact with spike of SARS-CoV-2 and facilitate viral intery to the host Increased sACE2 has correlation with inflammatory response and endothelial dysfunction	Controversial	[70, 72, 73]
sRAGE	Is associated with inflammatory diseases, bacterial infection, and lung damage Diagnosis factor for ARDS	Adverse effect	[76–78]
suPAR	Is involved in plasminogen activation pathway, regulation of cell adhesion, and proliferation and migration by interacting with extracellular matrix proteins Increased in infection and inflammatory conditions like arthritis, HIV infection Cause immune response activation Level of suPAR has positive correlation with severity and mortality of HIV and SARS-CoV-2	Adverse effect	[86–89]

sFlt-1, soluble fms-like tyrosine kinase-1; sACE2, soluble angiotensin-converting enzyme 2; sRAGE, soluble receptor for advanced glycation end products; suPAR, soluble urokinase-type plasminogen activator receptor; HIV, human immune-deficiency virus; ARDS, acute respiratory distress syndrome

### Soluble fms-like tyrosine kinase-1 (sFLT-1)

Vascular Endothelial Growth Factor (VEGF) and its associated family members regulate angiogenesis primarily through the engagement of the VEGF receptor 1, also referred to as fms-like tyrosine kinase 1 (Flt1) and VEGF receptor 2 molecules. The soluble form of Flt1 is created by alternatively spliced mRNA that binds and inhibits VEGF and placental growth factor (PlGF) signaling [69]. The excess level of sFlt-1 has been demonstrated to induce endothelial dysfunction, particularly in pre-eclampsia [70, 71]. In previous studies, sFlt-1 was identified as a biological marker of endothelial dysfunction associated with bacterial sepsis and severity [72], and it was increased in COVID-19 patients [69, 73]. The higher circulating level of sFlt-1 in COVID-19 identifies patients with a severe condition related to endothelial dysfunction and respiratory failure [72–74]. In this context, the application of sFlt-1 as an indication of endothelial dysfunction could offer a new strategy for diagnosing and treating COVID-19 patients.

### Soluble Angiotensin-converting enzyme 2 (sACE2)

Angiotensin-converting enzyme 2 (ACE2) is the main receptor for SARS-CoV-2 entry into cells. ACE2 is a crucial component of the classical renin-angiotensin system (RAS), which counterbalances the detrimental effects of angiotensin II (Ang II)/angiotensin II receptor type 1, including pro-inflammatory, prothrombotic, proliferative, and vasoconstrictive effects through catalytic cleavage of Ang II [75]. ACE2 can also be shed from the cell surface in soluble form (sACE2) with preserving its catalytic activity. It's possible during viral infections that when viral glycoprotein binds to ACE2, the shedding of ACE2 induces [76]. Yeung et al. confirmed that sACE2 or sACE2 vasopressin could interact with the spike protein of SARS-CoV-2 and facilitate virus cell entry through receptor endocytosis (AT1 or AVPR1B, respectively) [77]. After virus-induced shedding, an elevated level of sACE2 has been revealed in COVID-19 patients. Among these patients, sACE2 displayed correlations with inflammatory response markers and endothelial dysfunction, suggesting a connection with various cell injuries or release from different cell types [75, 78]. In contrast, another study reported a reduced level of sACE2 in patients. Based on this finding, sACE2 is likely to play protective effects in patients with COVID-19 [79]. In line with this, human recombinant sACE2 has been investigated as a potential treatment for SARS-CoV-2 infection patients and was correlated with a reduction in the level of cytokines involved in SARS-CoV-2 pathology angiotensin II and viral loads [80]. These contradictory results may be due to differences in the sample sizes of studies. However, evaluating sACE2 concentration and its role in COVID-19 patients’ needs further investigation.

### Soluble receptor for advanced glycation end products (sRAGE)

The receptor for advanced glycation end products (RAGE) is involved in the immune responses to infection, inflammation, and thereby endothelial damage. Alveolar epithelial cells in the lungs are the major sites of RAGE expression [81]. Its soluble form (sRAGE) can be produced by cleavage of the transmembrane RAGE or alternative mRNA splicing and acts as a competitive inhibitor of RAGE-mediated signaling [81]. The circulating sRAGE levels are associated with increased inflammatory diseases, bacterial infections, and lung damage [82, 83]. Also, its plasma levels predict the development of ARDS in high-risk ICU patients [84]. So, a higher level of sRAGE in influenza A virus pneumonia [85] and lung injury were indicated [86]. Studies revealed that the concentration of sRAGE increased in cases with severe COVID-19 [87, 88]. The elevated levels of sRAGE were also observed in COVID-19 patients who are diabetic or non-diabetic [89]. In addition, it was found that a high level of sRAGE could help predict respiratory failure, mechanical ventilation needs, and mortality rate in COVID-19 patients [88]. Moreover, studies conducted by Calfee et al. in lung transplantation have revealed a positive correlation between sRAGE levels and hospitalization duration [90]. It suggests sRAGE as an essential biomarker in COVID-19 so that monitoring serum levels of sRAGE can help improve the patient's care in the ICU. That sRAGE modulation may also be a therapeutic option for patients with COVID-19.

### Soluble urokinase plasminogen receptor (suPAR)

The urokinase-type plasminogen activator receptor (uPAR) as a part of the uPA system is predominantly expressed by endothelial cells, activated T lymphocytes, monocytes, and macrophages. In addition, the soluble form of uPAR (suPAR) results from the cleavage of the membrane-bound uPAR and is detectable in body fluids [91]. suPAR and its ligands have been implicated in several physiological and pathological processes, including the plasminogen activation pathway, regulation of cell adhesion, and proliferation and migration by interacting with extracellular matrix proteins [92]. In this regard, under inflammatory and infectious conditions, such as arthritis and HIV, the serum level of suPAR is elevated, indicating the activation of the immune response [93]. Researchers have found a strong correlation between suPAR levels in serum and severity of infection and mortality in patients with HIV infection [94]. In addition, recently, an increased level of sACE2 in COVID-19 patients and its correlation with the severe form of the disease has been reported [95]. In contrast to other biomarkers, such as IL-6, CRP, D-dimers, and ferritin, suPAR increases earlier in COVID-19 patients, reflecting a higher risk of disease progression to respiratory failure and respiratory failure mortality [91, 96, 97]. It suggests more attention to patients with a high level of suPAR to identify disease progression early may greatly facilitate the management of COVID-19 patients.

## Conclusion and future perspectives

**Fig. 3:**
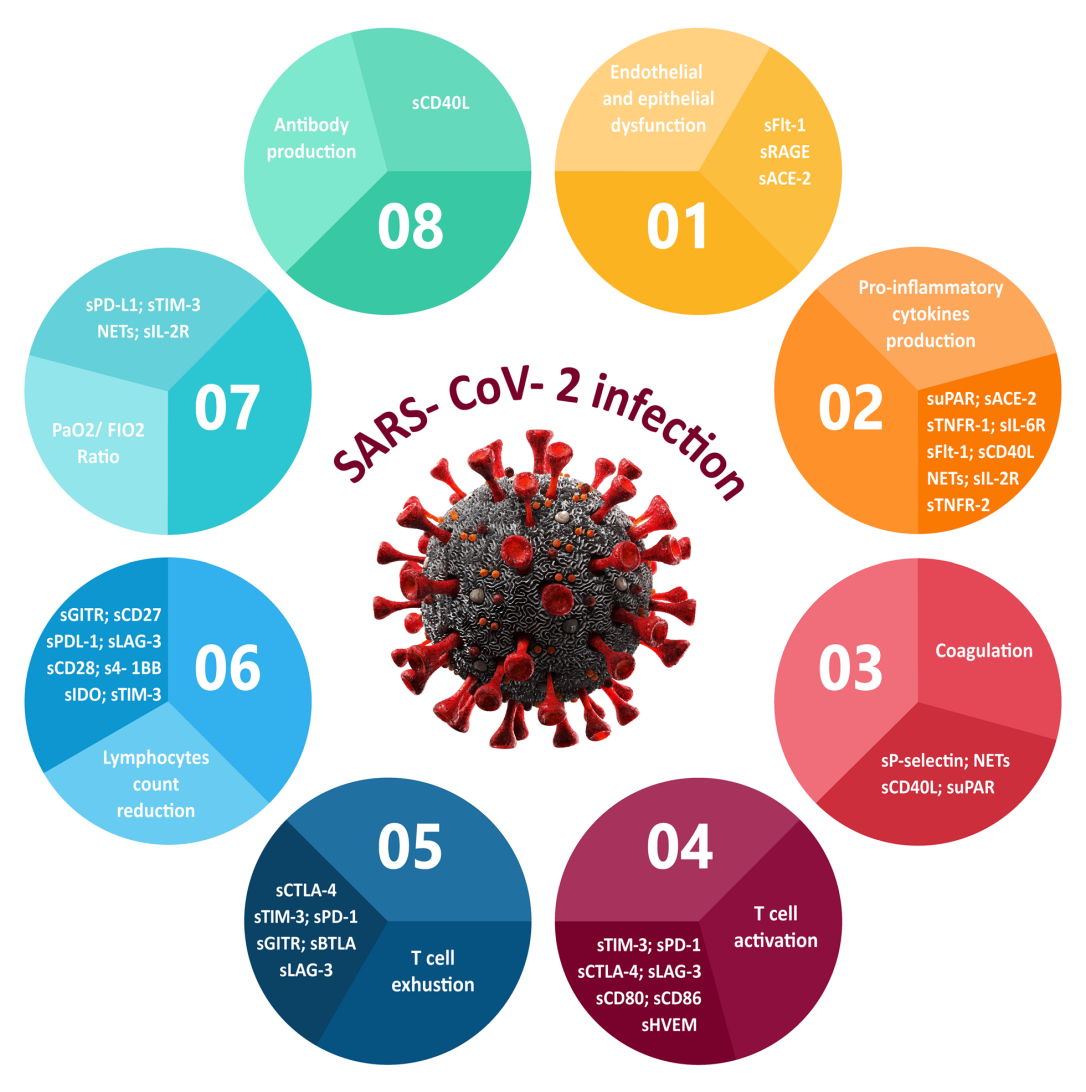
Overview of SARS-CoV-2 infection effects on the immunological and physiological processes of the body via soluble mediators

Efficacious responses against viral infections, which mostly depend on the regulation of immune responses, are crucial for the successful recovery of COVID-19 patients. Among the major research topics in recent years, soluble immune mediators have essential roles in regulating the immune system in the state of health and disease. However, their exact mechanisms of action are not fully understood. Several investigations have been conducted on the correlation of soluble immune checkpoints, receptors, and other mediators with SARS-CoV-2 infection. Based on this finding, during SARS-CoV-2 infections, dysregulation in the concentration of such molecules with an effect on immune responses can lead to disease pathology and play a significant role in COVID-19 severity and its associated mortality rate. These findings provide the first insights into them as essential factors for evaluating during SARS-CoV-2 infections. Figure 3 summarizes the immunological and physiological processes of the body via soluble mediators following SARS-CoV-2.

Modulation of their circulating levels may be a therapeutic option for COVID-19 patients. Further research is required to prove that targeting these soluble mediators ameliorates the severity of the disease. It has been suggested that immune checkpoint inhibitors may be valuable for treating several infectious diseases including HIV and HBV [98] and some of them like anti-PD-1, anti-PD-L1, and CTLA-4-Ig are approved for treatment of some cancers and autoimmune diseases. Recently, anti-PD-1 has been studied in a number of clinical trials for patients with COVID-19 (NCT04343144, NCT04268537, and NCT04356508) but the results of these studies have not been published to date. However, it can be challenging to find more specific antibodies to distinguish between full-length receptors and their soluble form. Furthermore, manipulating such mediators could provide a novel therapeutic target for patients by regulating the immune system. In this regard, antibodies that target these molecules specifically can be used to neutralize their effects in the progression of the disease.

## Data Availability

Not applicable.

## Abbreviations

COVID-19: Coronavirus disease 2019
SARS-CoV-2: Severe acute respiratory syndrome coronavirus type 2
ARDS: Acute respiratory distress syndrome
ILs: Interleukins
PD-1: Programmed death 1
IL-6: Interleukin 6
IFNs: Interferon's
PD-1: Programmed cell death protein 1
PD-L1: Programmed death-ligand 1
Foxp3: Forkhead box P3
PaO2: Partial pressure of oxygen
FIO2: Fraction of inspired oxygen
CRP: C reactive protein
sTim-3: Soluble T-cell immunoglobulin domain and mucin domain 3
NT-proBNP: N-terminal pro-B-type natriuretic peptide
sCTLA-4: Soluble cytotoxic T-lymphocyte antigen 4
sLAG-3: Soluble lymphocyte-activation gene 3
sGITR: Soluble glucocorticoid-induced TNFR-related protein
sBTLA: Soluble B and T lymphocyte attenuator
sHVEM: Soluble herpes virus entry mediator; TNFRSF14
sIDO: Soluble indoleamine 2,3-dioxygenase
s4-1BB: Solubl tumor necrosis factor ligand superfamily member 9 (sTNFSF9)
CDs: Cluster of differentiation
TNF-Rs: Tumor necrosis factor receptors
ADAM17: A disintegrin and metalloprotease 17
NETs: Neutrophil extracellular traps
PMNs: Polymorphonuclear
MPO: Myeloperoxidase
sP-selectin: Soluble platelet activation markers
sFLT-1: Soluble fms-like tyrosine kinase-1
VEGF: Vascular Endothelial Growth Factor
Flt1: Fms-like tyrosine kinase 1
PlGF: Placental growth factor
sACE2: Soluble angiotensin-converting enzyme 2
RAS: Renin-angiotensin system
sRAGE: Soluble receptor for advanced glycation end products
suPAR: Soluble urokinase plasminogen receptor
NK cells: Natural killer cells
